# Circulating microRNA 134 sheds light on the diagnosis of major depressive disorder

**DOI:** 10.1038/s41398-020-0773-2

**Published:** 2020-03-16

**Authors:** Han-ping Zhang, Xiao-lei Liu, Jian-jun Chen, Ke Cheng, Shun-Jie Bai, Peng Zheng, Chan-juan Zhou, Wei Wang, Hai-yang Wang, Lian-mei Zhong, Peng Xie

**Affiliations:** 1 Department of Neurology, The First Affiliated Hospital of Chongqing Medical University, Chongqing, 400016 China; 2 NHC Key Laboratory of Diagnosis and Treatment on Brain Functional Diseases, Chongqing Medical University, Chongqing, 400016 China; 3 Department of Neurology, The First Affiliated Hospital of Kunming Medical University, Yunan, China; 4 Institute of Life Sciences, Chongqing Medical University, Chongqing, 400016 China; 5 Department of Laboratory, The First Affiliated Hospital of Chongqing Medical University, Chongqing, 400016 China; 6 Department of Neurology, Yongchuan Hospital of Chongqing Medical University, Chongqing, 400016 China

**Keywords:** Diagnostic markers, Molecular neuroscience

## Abstract

Major depressive disorder (MDD) is a prevalent and debilitating psychiatric mood disorder that lacks objective laboratory-based tests to support its diagnosis. A class of microRNAs (miRNAs) has been found to be centrally involved in regulating many molecular processes fundamental to central nervous system function. Among these miRNAs, miRNA-134 (miR-134) has been reported to be related to neurogenesis and synaptic plasticity. In this study, the hypothesis that plasma miR-134 can be used to diagnose MDD was tested. Perturbation of peripheral and central miR-134 in a depressive-like rat model was also examined. By reverse-transcription quantitative PCR, miR-134 was comparatively measured in a small set of plasma samples from MDD and healthy control (HC) subjects. To determine its diagnostic efficacy, plasma miR-134 levels were assessed in 100 MDD, 50 bipolar disorder (BD), 50 schizophrenic (SCZ), and 100 HC subjects. A chronic unpredictable mild stress (CUMS) rat model was also developed to evaluate miR-134 expression in plasma, hippocampus (HIP), prefrontal cortex (PFC), and olfactory bulb. We found that plasma miR-134 was significantly downregulated in MDD subjects. Diagnostically, plasma miR-134 levels could effectively distinguish MDD from HC with 79% sensitivity and 84% specificity, while distinguishing MDD from HC, BD, and SCZ subjects with 79% sensitivity and 76.5% specificity. Congruent with these clinical findings, CUMS significantly reduced miR-134 levels in the rat plasma, HIP, and PFC. Although limited by the relatively small sample size, these results demonstrated that plasma miR-134 displays potential ability as a biomarker for MDD.

## Introduction

Major depressive disorder (MDD) is a heterogenous disease, involving genetic and environmental factors. It has a lifetime prevalence of 16.6% (ref. ^1^), and commonly results in negative sequelae ranging from impaired psychosocial functioning to increased suicidality^2^. Its exact pathogenesis is still unclear, although there are many theories attempting to explain it^3–6^. Meanwhile, its diagnosis still relies on subjective assessment of patient symptoms^7^, rather than on objective laboratory-based testing, although much work has been done to identify biomarkers for its objective diagnosis^8–10^. This may lead to underdiagnosis and misdiagnosis in primary care settings, where general practitioners make routine diagnoses of depression^11^. Hence, the determination of biomarkers for MDD is necessary to increase diagnostic efficiency and facilitate patient care.

As a current area of interest in molecular diagnostics research, microRNAs (miRNAs) have been recently shown to be effective circulating biomarkers for many diseases^12–16^. These small non-coding RNAs, which regulate the translation of many genes, are involved in various biological processes in the central nervous system^17^. Scientific interest in identifying miRNAs’ relevance to the pathogenesis of prevalent mental disorders has gained momentum^18–20^, indicating that miRNAs have a strong potential to contribute to a better understanding of the pathophysiology underlying MDD. Compared to our group’s metabolomic^21^ and proteomic^22^ approaches, a miRNA-based approach could display advantages in body fluid sampling^23^ and robust stability^24^. Arguably, it is more clinically practical to detect plasma miRNAs as opposed to plasma metabolites and proteins.

The Pubmed literature search was performed to select miRNAs associated with depression. Finally, miRNA-134 (miR-134) successfully draw our attention. Previous study reported that miR-134 has stage-specific effects on cortical development^25^; furthermore, it localizes to the synapto-dendritic compartment of rat hippocampal neurons and is closely related to activity-dependent dendritic outgrowth, synaptic transmission, and synaptic plasticity^26,27^. Given miRNA-134’s important roles in multiple steps of synaptic development and plasticity regulation, and MDD’s association with abnormalities in synaptic function and neurogenesis^28^, we chose miRNA-134 as a potential biomarker for diagnosing MDD. At such a low plasma concentration, miR-134 levels were too low for detection. Therefore, the reverse-transcription quantitative PCR (RT-qPCR) technology with the advantage of unparalleled sensitivity was applied here to detect the plasma miR-134 levels^29^.

**Fig. 1:**
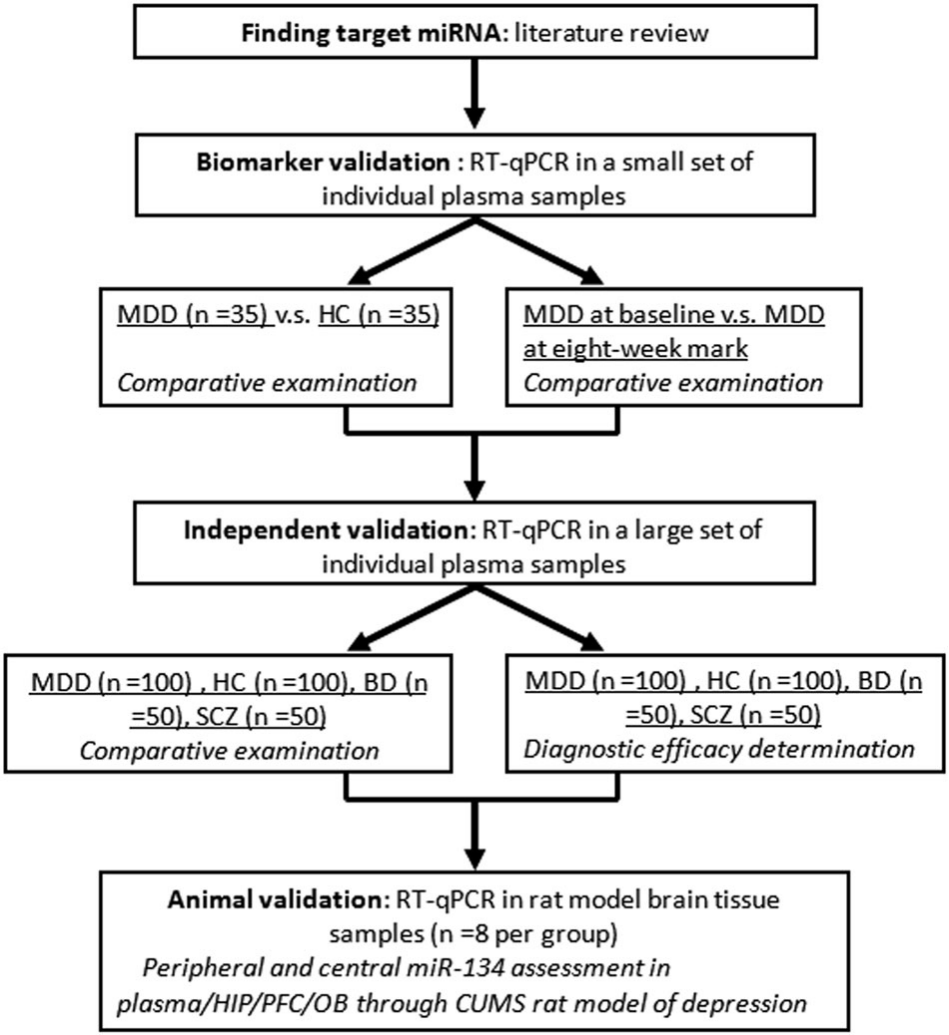
Flow chart of the experimental procedures. To identify a potential miRNA for diagnosing MDD, we firslty conducted literature review to find the target miRNAs. Then, we used a small set of samples to vaildate the target miRNAs. Finally, we used a large set of samples to independently validate the identified miRNA. Meanwhile, this miRNA was also validated in brain tissue of rat model.

To assess plasma miR-134’s ability as a biomarker for MDD, a multiphase study was designed (Fig. 1). The first step was identifying the differential expression of plasma miR-134 in MDD, and, more profoundly, determining whether this alteration reverses upon symptomatic improvement. Peripheral miR-134 levels were also comparatively analyzed in a large set of plasma samples from MDD, bipolar disorder (BD), schizophrenia (SCZ), and healthy control (HC) subjects to determine whether miR-134 can serve as an effective diagnostic biomarker for MDD. Through a chronic unpredictable mild stress (CUMS) rat model of depression, miR-134 levels in plasma and brain tissue—including hippocampus (HIP), prefrontal cortex (PFC), and olfactory bulb (OB)—were investigated to determine the relationship between peripheral and central miR-134 expression.

## Materials and methods

### Patient enrollment

The MDD, BD, and HC subjects were recruited from the First Affiliated Hospital of Chongqing Medical University, and SCZ subjects were enrolled from Chongqing Municipal Mental Hospital. All samples were collected between August 2009 and November 2011. MDD, BD, and SCZ subjects were diagnosed by two licensed psychiatrists independently according to DSM-IV criteria and disease severity was evaluated through the Hamilton Rating Scale for Depression (HAM-D, 17-item version)^30^, the Beck Depression Inventory (BDI, 13-item version)^31^, the Beck-Rafaelsen Manic Scale^32^, and the Positive and Negative Syndrome Scale^33^, respectively. Only depressed candidates with HAM-D scores > 17 were recruited into the MDD cohort. All candidates with one or more confounding factors, namely preexisting physical or other mental disorders, medication, and/or illicit drug use were excluded. During the same time period, HC subjects were recruited from the Medical Examination Center of the First Affiliated Hospital. Candidates with any current or previous lifetime history of neurological, DSM-IV axis I/II, or systemic medical disorder were excluded. The Ethical Committee of Chongqing Medical University approved the protocols of this study and the procedures employed for sample collection. Informed written consent was obtained from all participants.

In the primary phase, biomarker validation was performed on plasma samples from 35 currently depressed MDD subjects, as well as their blood samples after an 8-week medication period, and 35 sex- and age-matched HC. The information of these subjects was described in Supplemental Table 1. In the independent validation phase, analysis was done on plasma samples from four cohorts: (i) MDD (*n* = 100), (ii) BD (*n* = 50), (iii) SCZ (*n* = 50), and (iv) HC (*n* = 100). The MDD, BD, and SCZ subjects were either undergoing antidepressant/antipsychotic treatments or were drug free at the time of sample collection. Justification of the sample-size determination is provided in Supplemental Table 2.

### Human plasma samples

Whole blood samples (2.5 ml per subject) were collected via venipuncture into EDTA-containing tubes (BD Biosciences) and processed for plasma isolation within 2 h post collection. To obtain plasma, the whole blood samples were centrifuged at 1000 × *g* for 15 min, and then the supernatants were transferred into RNase-free tubes, and stored at −80 °C prior to RNA extraction.

### Animal and CUMS procedure

For the CUMS study, 16 healthy adult male Sprague-Dawley rats (weights: 230–280 g; age: 3–4 months) were purchased from the animal facility of Chongqing Medical University, were used. The rats were kept under standard conditions (12 h light/dark cycle; lights on at 7:00 a.m.; 22 ± 1 °C ambient temperature; 52 ± 2% relative humidity; and food and water ad libitum), unless otherwise stated. After adaptation to laboratory conditions (7 days prior to the start of the experimentation), the rats were randomly divided into two groups: (i) those kept under CUMS conditions (CUMS, *n* = 8) and (ii) non-stressed controls (CON, *n* = 8). The sample size of rats in each group was estimated according to our previous study^6^. The CUMS procedure followed a previously described method^27^: each rat received one or two stressors per day, and the same stressor was not given in two consecutive days. At the end of CUMS procedure, the food and water were deprived for 24 h prior to behavioral testing, which was the final stressor in CUMS group. The rats in CON group were not disturbed and could ad libitum access to food and water. Meanwhile, to avoid any acoustic or olfactory communication, they were isolated from the rats in CUMS group. The study was approved by the Ethics Committee of Chongqing Medical University, and all procedures were in accordance with NIH Guidelines for Animal Research. Special care was taken to minimize the number and suffering of animals.

### Behavioral tests

All rats were placed in the testing room 30 min prior to behavioral testing. All tests took place in a soundproof room between 8:00 a.m. and 1:00 p.m. After each test, rats were returned to their home cages and then to the holding room once all animals were tested. The open field test (OFT) was performed to measure spatial exploration behavior in rodents. The sucrose preference test (SPT) was conducted as a measure for the anhedonic effect of CUMS (ref. ^34^). The detailed information of OFT and SPT was described in Supplemental File 1.

### Rat plasma and tissue samples

Under anesthesia by chloral hydrate overdose, rats (CUMS group (*n* = 8) and CON group (*n* = 8)) were thoracotomized, and were immediately exsanguinated of 6–8 ml blood by heart puncture into EDTA-containing tubes (BD Biosciences). The plasma processing steps were the same as those for human plasma. Then, the rats were sacrificed by dislocation of the cervical vertebrae, and their whole brains were removed. The HIP, PFC, and OB were macrodissected from the whole brain, weighed, rapidly frozen with liquid nitrogen, and stored at −80 °C.

### RNA extraction

For all samples, total RNA was extracted using TRIzol reagent (Invitrogen Life Technologies). First, 0.75 ml or 1 ml TRIzol reagent was added to 0.25 ml plasma or 50–100 mg rat brain tissue samples. Samples were vortex mixed and allowed to stand for 5 min at room temperature; 200 μl of chloroform was then added to remove the proteins. The plasma and brain tissue samples were centrifuged at 12,000 × *g* for 15 min at 4 °C, supernatants were transferred to separate vials, and RNA was precipitated with 0.5 ml isopropanol. After incubation at room temperature for 10 min and centrifugation at 12,000×*g* for 10 min at 4 °C, the RNA pellet was rinsed, air dried, and resuspended in 20 μl RNAse-free water. Purified RNA samples were quantified with a NanoDrop ND-1000 spectrophotometer (Thermo Scientific) via absorbance measurements at 260 and 280 nm. The concentration of RNA extracted from plasma and tissue samples ranged from 10.04–30.32 ng/μl, and 405.18–1921.11 ng/μl, respectively.

### Real-time RT-qPCR

The study was conducted according to MIQE (minimum information for publication of quantitative real-time PCR experiments) guidelines (Supplemental File 1)^35^. The description of the genes, primers, amplicons, and other details were described in Supplementary Tables 3 and 4. Total RNA from each sample was reverse transcribed with a miRNA-specific stemloop reverse-transcriptase primer (Sangon Biotech Co. Shanghai, China); U6 RNA was used for normalization. The cDNA was amplified by real-time PCR with individual miRNA-specific primers (Sangon Biotech Co. Shanghai, China). The standard curves were described in Supplementary Table 5. All reactions were run in triplicate, and results were normalized to those of U6 RNA. The median in each triplicate was used to calculate relative miRNA concentrations. Expression fold changes were determined using the ΔCq method and reported as 2^−ΔΔCq^, where Cq is the quantification cycle^29^. RT-qPCR investigators were blinded to the grouping information. The repeatability (intra-assay variation) of qPCR measurements was described in Supplementary Tables 6 and 7. Details of the RT-qPCR methodology are provided in Supplemental File 1.

### Statistical analysis

Student’s *t*-test, paired *t*-test, Wilcoxon Mann–Whitney test, or *χ*^2^ test was used when appropriate. Receiver operating characteristic (ROC) curves were generated to assess the diagnostic accuracy of parameters. Spearman correlation analysis was performed to reveal the correlation between plasma miR-134 expression and depression severity. When appropriate, one-way analysis of variance was conducted. If a significant difference was found, a Bonferroni post hoc test or Tamhane’s T2 post hoc analysis was conducted to determine which groups differed significantly according to the equal variance criterion. Statistical analysis was performed on SPSS 19.0.

## Results

### Subject characteristics

Demographic and clinical characteristics of all recruited subjects were displayed in Table 1. The two cohorts in the primary phase were not statistically differentiable by any key demographic characteristic, while a portion of cohorts in the validation phase were not gender or age matched, suggesting the unbiased biomarker discrimination in the general population. All MDD subjects in both phases scored higher on the baseline HAM-D than both BD and HC controls. The 8-week follow-up HAM-D scores of MDD subjects in the primary phase significantly changed after antidepressant therapy. According to the HAM-D scores, follow-up MDD subjects were classified into three categories: (i) non-response (no clinical meaningful response post treatment), (ii) partial response (>25%, but <50% decrease, in HAM-D score), and (iii) response (≥50% decrease in HAM-D score with a final HAM-D score of ≤15)^36^. The antidepressant drugs can be classified into five types: (i) heterocyclics/TCAs, (ii) SSRIs, (iii) SNRIs, (iv) MAOIs, and (v) atypical (mixed action) antidepressants. The distribution of antidepressant treatments dosed before enrollment and during the 8-week study period was shown in Supplemental File 1. Among all subjects in the primary phase, 15 subjects were drug free, and 20 subjects were receiving a single or combination antidepressant therapy at enrollment.

**Table 1:** Demographic and clinical features of participants.

Primary phase					
Variable^a^	HC	MDD at baseline	*P*-value (HC vs. MDD)	MDD at 8-week mark	*P*-value (baseline vs. 8-week mark)
Sample size^b^	35	35	–	35	–
Sex (M/F)^c^	13/22	10/25	0.445	10/25	–
Age (year)^b^	34.4 ± 10.5	36.9 ± 12.7	0.378	36.9 ± 12.7	–
BMI^b^	23.3 ± 3	22.9 ± 3.2	0.563	22.5 ± 3	0.606
HAM-D scores^b^	0.9 ± 1	23.4 ± 3.7	<0.001	12.9 ± 6.4	<0.001
BDI scores^b^	0.4 ± 0.6	19.6 ± 6	<0.001	12.1 ± 8	<0.001
Independent validation phase					
Variable	HC	MDD	BP	SCZ	
Sample size	100	100	50	50	
Sex (M/F)	43/57	37/63	24/26	14/36	
Age (year)	32.4 ± 11.8	35.9 ± 11.5	29.8 ± 13.5	42.6 ± 11.2	
BMI	23.1 ± 2.9	22.6 ± 3.2	22.3 ± 3.3	22.8 ± 3	
HAM-D scores	0.7 ± 0.9	22.9 ± 3.8	13.6 ± 9.5		
BRMS scores			6.2 ± 8.1		
PANSS scores					59 ± 14.5

*HC* healthy control subjects, *MDD* major depression disorder subjects, *BP* bipolar disorders subjects, *SCZ* schizophrenia subjects, *M* male, *F* female, *BMI* body mass index, *HAM-D* Hamilton depression rating scale, *BDI* Beck Depression Inventory, *BRMS* Beck-Rafaelsen Manic Scale, *PANSS* Positive and Negative Syndrome Scale.

^a^Data of age, BMI, HAM-D scores, and BDI scores are presented as mean ± (SD)s.

^b^Student’s *t*-test.

^c^Two-sided Chi-squared test.

### Plasma miR-134 downregulation in MDD

**Fig. 2:**
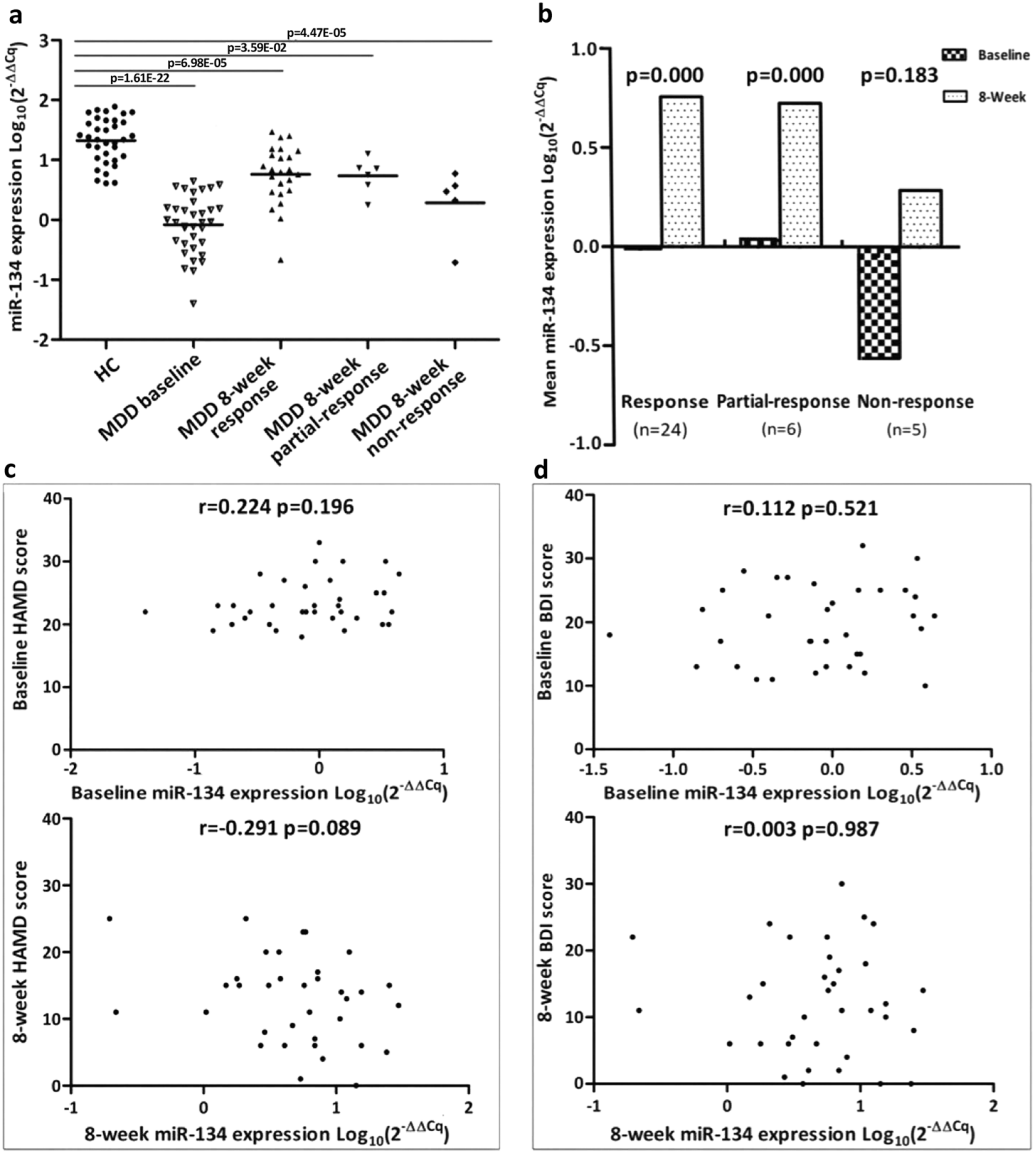
Plasma miR-134 levels from HC and MDD subjects at baseline and 8-week follow-up. **a** Plasma miR-134 levels in HC and MDD (HC vs. MDD baseline (*p* = 1.61E−22), HC vs. MDD 8-week response (*p* = 6.98E−05), HC vs. MDD 8-week partial response (*p* = 3.59E−02), and HC vs. MDD 8-week non-response (*p* = 4.47E−05)). **b** Plasma miR-134 levels of response (*n* = 24), partial response (*n* = 6), and non-response (*n* = 5) MDD subjects at baseline and 8-week follow-up. **c** Correlations between plasma miR-134 levels and HAM-D scores at baseline and 8-week follow-up for MDD subjects. **d** Correlations between plasma miR-134 levels and BDI scores at baseline and 8-week follow-up for MDD subjects.

The differential expression of plasma miR-134 was assessed in MDD and HC subjects. Relative concentrations of plasma miR-134 in MDD and HC were shown (Fig. 2a); baseline plasma miR-134 levels of MDD subjects were significantly lower than those of HC subjects (*p* = 1.61E−22). Plasma miR-134 levels did not differ between drug-free and drug-treated subjects at baseline when the subjects first presented with significant depressive symptoms (*p* = 0.785).

To determine whether plasma miR-134 downregulation corrects upon symptomatic improvement, its level was measured at 8-week follow-up (Fig. 2b). After 8 weeks of antidepressant therapy, mean plasma miR-134 levels did not significantly change in non-response MDD subjects (*p* = 0.183). In the partial-response and response MDD subjects, mean plasma miR-134 levels were significantly higher than baseline. But, there were still significant decreases in plasma miR-134 levels across all three MDD groups as compared to HC subjects. No correlation between plasma miR-134 levels and HAM-D (Fig. 2c) or BDI (Fig. 2d) scores was observed in MDD subjects at enrollment (Spearman *r* = 0.224, *p* = 0.196; and *r* = 0.112, *p* = 0.521, respectively), or at 8-week follow-up (Spearman *r* = −0.291, *p* = 0.089; and *r* = 0.003, *p* = 0.987, respectively).

### Independent validation of miR-134

**Fig. 3:**
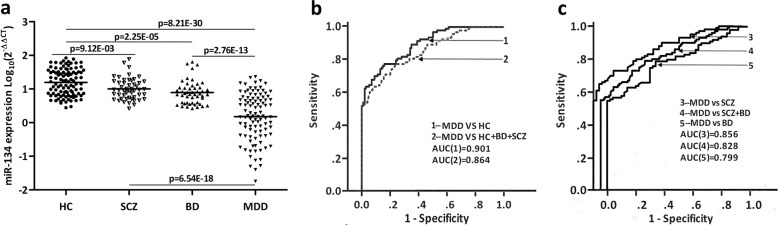
Plasma miR-134 levels in HC, SCZ, BD, and MDD, and ROC curve analysis for plasma miR-134 in MDD. **a** Plasma miR-134 levels in HC (*n* = 100), MDD (*n* = 100), BD (*n* = 50), and SCZ (*n* = 50) subjects (HC vs. MDD (*p* = 8.21E−30), HC vs. BD (*p* = 2.25E−05), HC vs. SCZ (*p* = 9.12E−03), MDD vs. BD (*p* = 2.76E−13), and MDD vs. SCZ (*p* = 6.54E−18)). **b** ROC curve analysis on plasma miR-134’s ability to differentiate MDD (*n* = 100) from HC (*n* = 100) and BD + SCZ + HC (*n* = 200) subjects. **c** ROC curve analysis on plasma miR-134’s ability to differentiate MDD (*n* = 100) from BD (*n* = 50), SCZ (*n* = 50), and BD + SCZ (*n* = 100), respectively.

The miR-134 levels were measured in plasma samples from four cohorts: MDD, BP, SCZ, and HC. Compared to HC, circulating levels of miR-134 were significantly lower in the MDD (*p* = 8.21E−30), BD (*p* = 2.25E−05), and SCZ cohorts (*p* = 9.12E−03). Plasma miR-134 levels of both the BP and SCZ cohorts were significantly higher relative to the MDD cohort (*p* = 2.76E−13, *p* = 6.54E−18; Fig. 3a).

To determine the diagnostic accuracy of plasma miR-134 levels, ROC curve analysis was conducted (Fig. 3b, c). When the MDD and HC cohorts were compared, the AUC was 0.901 (95% CI, 0.861–0.941). Using 5.99 as a cutoff value for the relative expression level, the sensitivity and specificity of diagnosing MDD were 79% and 84%, respectively. When the MDD and BP cohorts were compared, the AUC was 0.799 (95% CI, 0.731–0.867). When the MDD and SCZ cohorts were compared, the AUC was 0.856 (95% CI, 0.799–0.914,). With the same cutoff value of 5.99, the sensitivity and specificity of diagnosing the MDD cohort against the BP and SCZ cohorts were 79% and 69%, respectively. Plasma miR-134 distinguished the MDD from the HC plus the BD and SCZ with an AUC of 0.864 (95% CI, 0.799–0.914), translating to a 79% sensitivity and 76.5% specificity.

### Mir-134 downregulation in CUMS rat model

**Fig. 4:**
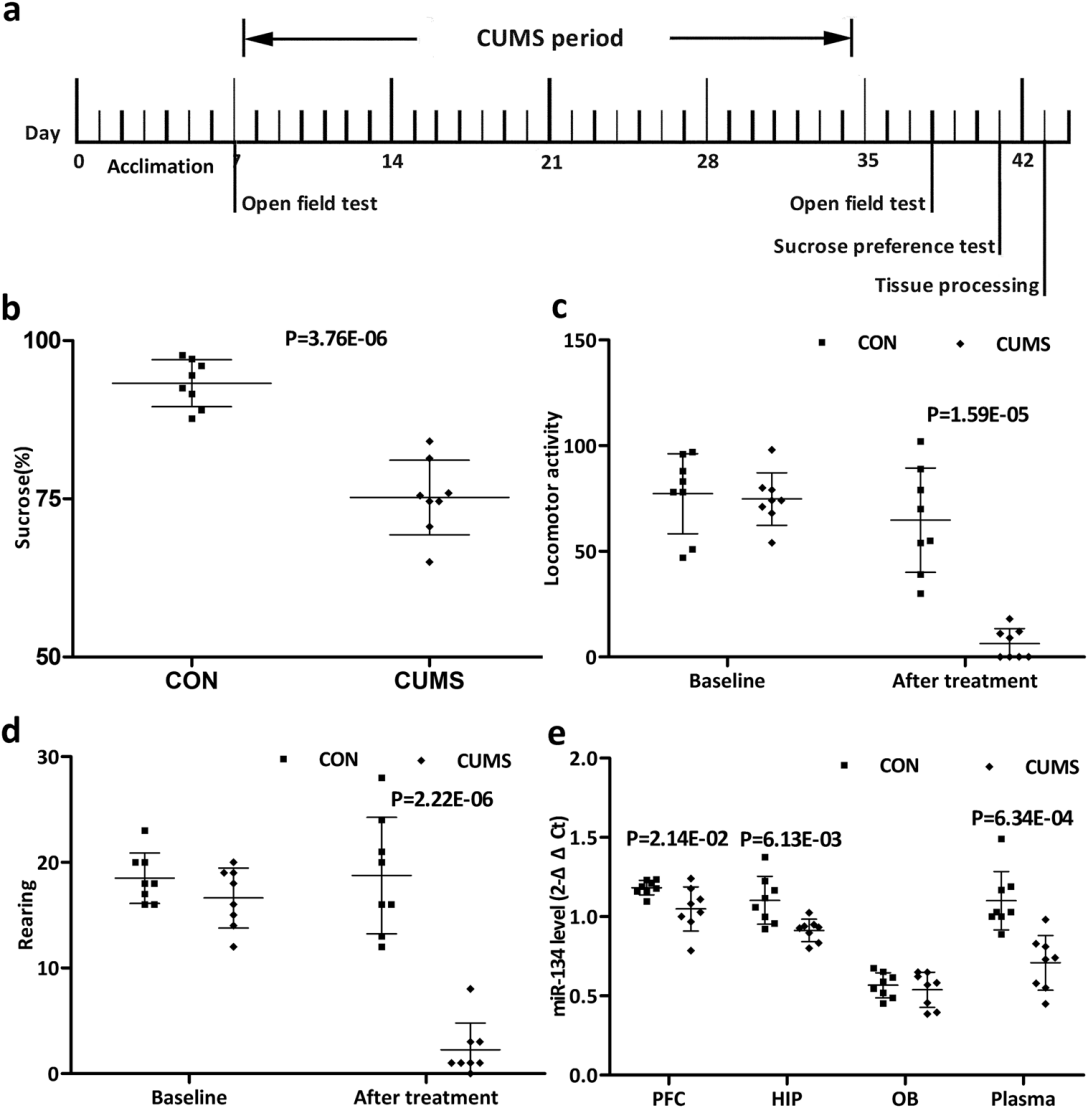
Procedural time schedule, CUMS rat model quality, and rat miR-134 levels. **a** Time schedule (in days) for the various CUMS rat model procedures. Open field testing was conducted on days 7 and 38. Sucrose preference testing was completed at day 41. **b** Results of sucrose preference testing. **c** Number of locomotor activities at baseline and after chronic stress. Data are presented as means ± SDs (*n* = 8 per group). **d** Number of rearings at baseline and after chronic stress. **e** Effect of chronic stress on miR-134 levels in the rat plasma, PFC, HIP, and OB.

To determine whether the peripheral miR-134 alteration linked to the perturbation of miR-134 in corticolimbic structures involved in emotional and mood regulation, plasma miR-134 levels and central miR-134 levels in the HIP, PFC, and OB were assessed through a CUMS rat model. The time schedule was provided in Fig. 4a. To evaluate model quality, the OFT scores and SPT percentages were used. Data showed significant differences between the CUMS (*n* = 8) and CON (*n* = 8) groups in both parameters (Fig. 4b, d), indicating CUMS’s efficiency in inducing a depressive-like status. RT-qPCR analysis demonstrated that 4 weeks of CUMS significantly reduced miR-134 levels in plasma (*p* < 0.001), the HIP (*p* < 0.01), and the PFC (*p* < 0.05). However, miR-134 levels were not significant changed in the OB (*p* = 0.565; Fig. 4e).

## Discussion

Although many studies have been undertaken to identify potential MDD biomarkers, no definitive biomarkers have yet been established. Several studies have shown decreased circulating BDNF levels in MDD patients; however, an evident overlap in BDNF levels between MDD and HC subjects exists^37–39^. On a genetic/epigenetic level, Fuchikami et al.’s analysis of genomic DNA from peripheral blood indicated that CpG I DNA methylation profiles of the BDNF gene might serve as a diagnostic biomarker for MDD; however, its sample size was relatively small^40^. In light of the present situation, it is important to develop a highly sensitive diagnostic biomarker for MDD.

This study is the first to clinically establish altered expression of peripheral miR-134 in MDD patients, while simultaneously demonstrating altered peripheral and central miR-134 in a depressed-like animal model. We found that: (i) plasma miR-134 downregulation is present during the acute depressive phase of MDD independent of antidepressant usage; (ii) plasma miR-134 levels increased in conjunction with symptomatic improvement after an 8-week antidepressant treatment period; and (iii) subjects with the lowest concentrations of miR-134 might be non-responsive to conventional antidepressants (Fig. 2b). These facts indicate that miR-134 is a state-dependent biomarker unrelated to pharmacotherapeutic status, and a greater extent of miR-134 downregulation indicates a poorer prognosis in response to conventional pharmacotherapy.

Considering that MDD, BP, and SCZ share some similar symptoms and pathophysiological changes, plasma miR-134 levels in 50 BP and 50 SCZ subjects were examined to validate miR-134’s use in differential diagnosis. Consistent with previous studies on miR-134 in bipolar mania^41^ and SCZ (ref. ^42^), compared to HC subjects, circulating levels of miR-134 were significantly lower in BP and SCZ subjects. However, MDD displays the most significant downregulation of plasma miR-134 levels. Diagnostically, the plasma miR-134 level effectively distinguish MDD subjects from HC with a 79% sensitivity and 84% specificity, while distinguishing MDD subjects from HC plus BD and SCZ subjects with a 79% sensitivity and 76.5% specificity.

The peripheral bloodstream, as a “sentinel tissue”^43^, is linked to other tissues that continually diffuse into the circulation. To determine the relationship between peripheral and central miR-134, plasma and brain miR-134 levels were assessed through a CUMS rat model of depression. CUMS is an informative model in which to study depression, as the model mimics the role of socio-environmental stressors in precipitating a depressive-like pathology^44^. In concordance with the peripheral miR-134 downregulation found in MDD patients, miR-134 downregulation was found in the plasma as well as the HIP and PFC of the CUMS rat model. These results agree with a previous study reporting that miR-134 downregulation in the rat HIP CA1 and central amygdala in response to chronic stress^45^. The combined human and rat findings support miR-134’s candidacy as an effective peripheral diagnostic target for MDD.

MiR-134 is involved in several pathways regulating dendritic spine development and plasticity. A recent study reported that miR-134 expression in both murine and human hippocampi was upregulated in epilepsy relative to HC (ref. ^46^). In contrast, this study demonstrated that peripheral miR-134 expression in MDD patients, and both central and peripheral miR-134 expression in a depression-like rat model were both significantly downregulated. A key miR-134 target is cAMP response element-binding protein (CREB)^26^, which plays a key role in the activity-dependent regulation of BNDF expression. Hippocampal BDNF expression and CREB activity have both been shown to be upregulated in epilepsy and downregulated in stress and depression^47,48^. Synthesizing these findings, we speculated that miR-134 upregulation in epilepsy, by restricting CREB translation, serves as a posttranscriptional control element counteracting the BDNF upregulation. In MDD, the converse is in effect: miR-134 downregulation in MDD subjects serves to increase CREB translation in the context of decreased BDNF levels and CREB activity. Further research into this putative feedback loop is required to further elucidate miR-134’s role.

Several limitations should be mentioned here. Firstly, the subjects were from the same city, which might limit the general applicability of these findings^49,50^. Secondly, the number of subjects in this study was relatively small; but the high AUC values demonstrated the potential clinical applicability of miR-134 in diagnosing MDD. Thirdly, patients in the MDD, BD, and SCZ patient groups had the significantly lower plasma miR-134 levels than HC. Thus, it will not very clearly predict depression in a single patient or confine to other psychiatric diseases. Future studies were needed to identify the cutoff value of plasma miR-134 level that could be used to separate MDD from other psychiatric diseases, such as BD and SCZ. Fourthly, we did not control other parameters (e.g., measurement of corticosterone and adrenal gland weight) to validate the biological effects and relevance of their stress model. Fifthly, we did not collect data regarding CREB activity in rat specimen; the relationship between miR-134 and CREB was worthy of further study. Sixthly, we only considered a single miRNA as an adequate biomarker as opposed to a panel of miRNAs, especially considering the heterogeneity and complexity of MDD. Future studies should identify some new miRNAs as biomarkers for diagnosing MDD, and then assess whether or not the panel consisting of these new miRNAs and our miRNA-134 could significantly improve the diagnostic accuracy. Seventhly, we found that the plasma miRNA-134 levels did not correlate with the levels in PFC, OB, or HIP; then future studies were needed to find out whether or not we could use the peripheral miRNA expression as a proxy measure of brain miRNAs. Finally, other biosamples from MDD patients, such as cerebrospinal fluid, should be explored to ensure miR-134 being relevant to the pathogenesis of MDD. However, our study demonstrated that plasma miR-134 could be a potential biomarker in detecting and differentially diagnosing MDD, as well as a state marker for monitoring symptomatic improvement. Multicenter clinical trials were necessary to assess the feasibility of applying plasma miR-134 as biomarker for MDD.

## Supplementary information

Supplemental File
