# Supplementary material for: Circulating microRNA 134 sheds light on the diagnosis of major depressive disorder
Source: Transl Psychiatry. 2020 Mar 16;10:95. doi: 10.1038/s41398-020-0773-2 (PMC7075934; doi:10.1038/s41398-020-0773-2)
Supplement: Supplementary file 1 — Supplemental File [file 41398_2020_773_MOESM1_ESM.doc]

**Supplementary information**

**Table S1 Antidepressant medication distribution (%)^a^**

| Medication | Baseline (n=35) | Eight-week mark (n=35) |
| --- | --- | --- |
| Heterocyclics/TCAs | 0.0 | 0.0 |
| SSRIs | 40.0 | 74.3 |
| SNRIs | 5.7 | 8.6 |
| MAOIs | 0.0 | 0.0 |
| Atypical antidepressants^b^ | 28.6 | 65.7 |
| Hypnotics | 25.7 | 28.6 |
| Drug-free | 42.9 | 5.7 |

Abbreviations: TCAs, tricyclic antidepressants; SSRIs, selective serotonin reuptake inhibitors; MAOIs, monoamine oxidase inhibitors.

^a^Patients are only counted once for each medication category.

^b^Includes mirtazapine, trazodone, and flupenthixol/melitracen.

**Table S2 Results of prelimilary analysis:**

| T | D=1 | D=0 |
| --- | --- | --- |
| 1 | A1=16 | B1=17 |
| 0 | A2=4 | B2=3 |

Symbols: T, miR-134 test results (1 = positive, 0 = negative test); D, DSM-IV diagnostic results (1 = disease,0 = no disease). From this table, n=(A1+A2+B1+B2), SE^$^ = A1/(A1+A2), SP^$^ = B1/(B1+B2), and PD^$^=(A1+A2)/n. These data was used to estimated SE, SP and PD.

**Table S3 Details of MicroRNA 134**

| **MicroRNA ID** | **Sequence** | **miRBase Accession No.** |
| --- | --- | --- |
| hsa-miR-134 | UGUGACUGGUUGACCAGAGGGG | [MIMAT0000447](http://www.mirbase.org/cgi-bin/mature.pl?mature_acc=MIMAT0000447) **^a^** |
| rno-mir-134 | UGUGACUGGUUGACCAGAGGGG | [MIMAT0000840](http://www.mirbase.org/cgi-bin/mature.pl?mature_acc=MIMAT0000840) **^a^** |

**^a^** miRBase **(**<http://www.mirbase.org/>) Acc. No. of mature miRNA sequences alias v.19 (2012)

**Table S4 Measuring details of RT-qPCRs**

| **Gene symbol** | **RT primer sequences (5’---3’)** | **PCR primer sequences (5’---3’)** | **AT**  **(℃)** | **AL**  **(bp)** |
| --- | --- | --- | --- | --- |
| ***U6*** | CGCTTCACGAAT TTGCGTGTCAT | F:GCTTCGGCAGCACATATACTAAAAT  R:CGCTTCACGAATTTGCGTGTCAT | 60 | 89 |
| ***hsa-miR-134***  ***rno-mir-134*** | GTCGTATCCAGTGCGTGTCGTGGAGTCGGCAATTGCACTGGATACGACCCCCC | F:GGGTGTGACTGGTTGACC  R:CAGTGCGTGTCGTGGAGT | 60 | 65 |

AT: Annealing temperature; AL: Amplicon length.

**Table S5 Standard curves**

| **Gene** | **PCR-Efficiency** | **Slope** | **R^2^** | **y-Intercept** | **Dynamic range^a^** |
| --- | --- | --- | --- | --- | --- |
| **miR-134**^b^ | 2.005 | -3.311 | 0.9988 | 30.517 | 10.6-30.8 |
| **miR-U6** ^b^ | 1.946 | 3.450 | 0.9996 | 28.687 | 7.9-28.5 |

**^a^**Dynamic range represents the range of the Cq values between the highest and the lowest concentration of the generated standard curve.

^b^Amplicon dilutions; a dilution of 1x10^-6^ was defined as 1 arbitrary units. Amplicon dilutions were used for the generation of standard curves since cDNA dilutions were not available to obtain standard curves with a wide dynamic range.

**Table S6 Repeatability of qPCR measurements on miR-U6**

| **Dilution serials** | **(U6-1)Ct** | **(U6-2)Ct** | **(U6-3)Ct** | **U6 Average Ct** | **SD** | **S%** |
| --- | --- | --- | --- | --- | --- | --- |
| E6 | 8.416497 | 7.972785 | 8.3019705 | 8.2304175 | 0.23034747 | 2.798734 |
| E5 | 11.6073065 | 11.464015 | 11.677536 | 11.5829525 | 0.10882391 | 0.939518 |
| E4 | 14.602215 | 14.857192 | 14.805002 | 14.754803 | 0.13469696 | 0.912902 |
| E3 | 17.981157 | 18.414894 | 18.312822 | 18.236291 | 0.22677013 | 1.24351 |
| E2 | 22.03596 | 22.008074 | 21.912378 | 21.98547067 | 0.06481752 | 0.29482 |
| E1 | 25.44435 | 25.355953 | 25.10727 | 25.30252433 | 0.17477614 | 0.690746 |
| E0 | 28.072721 | 28.2645 | 27.743048 | 28.02675633 | 0.26374725 | 0.941055 |

SD=standard deviation; S%=SD x 100/mean

**Table S7** Repeatability of qPCR measurements on hsa-miR-134

| **Dilution serials** | **(hsa-miR-134-1)Ct** | **(hsa-miR-134-2)Ct** | **(hsa-miR-134-3)Ct** | **hsa-miR-134 Average Ct** | **SD** | **S%** |
| --- | --- | --- | --- | --- | --- | --- |
| E6 | 6.648224 | 6.4882016 | 6.6503487 | 6.595591433 | 0.09300839 | 1.41016 |
| E5 | 10.018263 | 9.991971 | 9.945431 | 9.985221667 | 0.03688211 | 0.369367 |
| E4 | 13.308339 | 13.233821 | 13.2550335 | 13.26573117 | 0.03839353 | 0.289419 |
| E3 | 16.428997 | 16.598087 | 16.524359 | 16.51714767 | 0.08477535 | 0.513257 |
| E2 | 19.772148 | 19.773785 | 19.841808 | 19.79591367 | 0.03975409 | 0.20082 |
| E1 | 22.833261 | 22.71441 | 22.595707 | 22.71445933 | 0.11877701 | 0.522914 |
| E0 | 26.029848 | 26.375517 | 25.51176 | 25.972375 | 0.43473716 | 1.673844 |

SD=standard deviation; S%=SD x 100/mean

**Supplementary Methods**

**1. Medication information**

The information of antidepressant medication distribution was desribeided in Table S1**.**

**2. Justification of the sample size**

To perform sample size calculations, sensitivity (SE), speciﬁcity (SP), and true prevalence of disease (PD) were assumed for the population; these were obtained by a [preliminary](http://dict.youdao.com/w/preliminary/) [analysis](http://dict.youdao.com/w/experiment/) of miR-134 level in the human plasma (n =20 in CON, n=20 in MDD) (Table S2). Subsequently, in the joint estimation approach, sample size N was chosen such that the joint 100(1-α)% conﬁdence region was acceptably small. The estimation approach was as follows^1^:


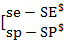
]^T^ V^-1^
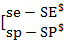
]=
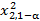


where the symbol
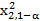
 denotes (1 −
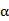
) quantile of the x^2^ distribution with 2 degrees of freedom, and the variance–covariance matrix V is as follows:


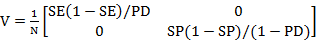


with SE, SP, and PD estimated, respectively, by SE^$^ = A1/(A1+A2), SP^$^ = B1/(B1+B2), and PD^$^=(A1+A2)/n, n=(A1+A2+B1+B2).

Finally, sample size calculations was performed. Assuming that the estimates (SE, SP and PD) equaled the spcified values (SE^$^, SP^$^, and PD^$^), and considering
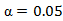
. For there was no striction criterion about how small of 95% confidence region was acceptably small, we treated the 95% confidence region of less than 0.3 as acceptably small. When N reached 90, the 95% confidence region of SP was more than 0.3; When N reached 100, the 95% confidence region of SE and SP were 0.25 and 0.27, respectively. Thus, N=100 per group was chosen as the final sample size.

**3. Behavioral tests**

Open field test (OFT): briefly, an apparatus consisting of a black square cage (100cm×100cm×40cm) was divided into 25×25cm^2^ equal squares on the arena floor. A single rat was placed in the center of the cage; after 30s of adaptation, behaviors including the number of locomotive movements (movement with all four paws) and the number of rearing movements (posture sustained with hindpaws on the floor) were recorded for 5min using a Sony DCR-SR45E camera located 190-200cm above the arena. The cage was thoroughly cleaned after each trial. Following this, OFT activity was scored manually from a computer screen by a blinded technician.

Sucrose preference test (SPT): rats were trained to adapt to a 1%(w/v) sucrose solution 72h prior to testing: two bottles of 1% sucrose solution were placed in each cage, and 24h later, 1% sucrose in one bottle was replaced with tap water for 24h. Post-adaptation, rats were deprived of water and food for 24h. This was immediately followed by the SPT, where all rats housed in individual cages had free access to two bottles: one containing 1% sucrose and the other tap water. The positions of the two bottles (left/right sides of the cages) were randomly varied. All fluid consumption was recorded by weighing the two bottles before testing and at 24h, and sucrose preference was assessed by the sucrose preference percentage (100%×sucrose consumption/(sucrose consumption + water consumption)).

**4. qPCR target information and qPCR Validation: Standard curves**

The description of the genes, primers, amplicons, and other details were described in Table S3 and Table S4. Standard curves were generated from diluted amplicons setting the undiluted sample as 1 arbitrary unit setting a dilution of 1x10^-5^ as 100 arbitrary units. The Cq values were calculated automatically by the ABI PRISM7900 software. Standard curves: the PCR-efficiency (E=10^-1/slope^) and the slope, intercept, and R^2^ as well as the so-called dynamic range were calculated by the ABI PRISM7900 software (Table S5). The repeatability (intra-assay variation) of qPCR measurements was described in Table S6 and Table S7.

**5. Stability validation of reference gene U6**

The small nucleolar RNA U6 has been widely used as references gene by virtue of its high expression and ubiquity. In plasma miRNAs analysis, U6 RNA has been commonly used^2-4^. However, some researches reported that the concentrations of U6 RNA is variable in serum and plasma samples^5^. To determine whether U6 is suitable for miRNA quantification by RT-qPCR in plasma samples, miRNAs microarray (n=5 per group) and RT-qPCR for U6 (totally n=30) were performd in plasma samples of MDD patients and healthy controls.

Firts, plamsa microarray data showed that U6 RNA was present in all examined 10 samples (ForeGround - BackGround > 100). The absolute fold change between the two groups was 1.017, and there was no significant difference (*P* = 0.79) between the two groups in microarray.

Second, in RT-qPCR for U6, Cq values were transformed into Q values according to the delta-Ct formula:

Q = E^delta-Cq^ = E^(minCq−sampleCq)^.

This method involves the choice of a calibrator sample having the lowest Cq value (minCq) of U6^6,7^. No Significant difference in the Q value (*P* = 0.388) of MDD group relative to the controls was observed for U6.

Third, the stability of U6 expression under the experimental conditions was evaluated by geNorm software using the improved version geNorm^Plus^ as an implementation of qBase^PLUS^ software (Biogazelle, Belgium)^8^. The geNorm M-value of U6 was lower than 0.15, indicating that the U6 expressions was relatively stable. Therefore, we concluded that U6 was suitable as reference gene to normalize miRNA RT-qPCR data under these experimental conditions.

**6. RT-qPCR METHODOLOGY**

cDNA synthesis of miRNAs: 200 ng of total RNA from each sample was reverse-transcribed with a miRNA-specific stemloop reverse-transcriptase primer (Sangon Biotech Co. Shanghai, China); U6 RNA was used for normalization. Each 20 µL reaction mix contained: 200 ng of total RNA, 2 μl of dNTP mix (2.5mM each, which contains dATP, dGTP, dCTP, and dTTP) (HyTest Ltd), 20 U Reverse Transcriptase (Epicentre), 0.5 uM microRNA specific stem-looped RT-primer (Sangon Biotech Co.), 8 U RNase Inhibitor (Epicentre), and 10 × RT buffer (Epicentre, which contains 250 mM Tris-HCl (pH 8.3), 200 mM KCl, 40 mM MgCl2, and 5 mM DTT). The cDNA synthesis was performed in Gene Amp PCR System 9700 (Applied Biosystems) with following steps: priming at 16°C for 30 min, transcription at 42°C for 40 min and enzyme inactivation at 85°C for 5 min. The cDNA samples were stored at -20°C until RT-PCR analysis.

qPCR of miRNAs: The qPCR measurements were carried out on the ABI PRISM7900 system (Applied Biosystems) in 384-well plates with a reaction volume of 10 ul and runs up to 40 cycles. The PCR reaction mixture of 10 µl contained: 2 μl of template cDNA, with 5 µl 2 × PCR Master Mix (Superarray), 1 µl miRNA-specific primers, and 2 µl RNase-free water. The cycling conditions were set as follows: initial activation of Taq polymerase at 95°C for 10 min followed by amplification steps – denaturation at 95°C for 10 sec, annealing/elongation at 60°C for 1 min with fluorescence acquisition. A melting curve was performed at the end of the PCR run over a range of 60–99°C, increasing the temperature stepwise by 2% every second. All non-template controls were negative (Cq > 35). Amplicon dilutions were used to generate calibration curves in order to obtain a wide dynamic range of miRNA measurements.

**References：**

1. Kosinski, A.S., Chen. Y., & Lyles, R.H. Sample size calculations for evaluating a diagnostic test when the gold standard is missing at random. Stat. Med. 2011;30:200.

2. Li, S. et al. Signature microrna expression profile of essential hypertension and its novel link to human cytomegalovirus infection. Circulation 2011;124:175-84.

3. Zhang, Y. et al. Plasma microrna-122 as a biomarker for viral-, alcohol-, and chemical-related hepatic diseases. Clin. Chem. 2010;56:1830-8.

4. Tijsen, A.J. et al. Mir423-5p as a circulating biomarker for heart failure. Circ. Res. 2010;106:1035-9.

5. Wang, K. et al. Comparing the microrna spectrum between serum and plasma. PLoS One 2012;7:e41561.

6. Livak, K.J., & Schmittgen, T.D. Analysis of relative gene expression data using real-time quantitative pcr and the 2(-delta delta c(t)) method. Methods 2001;25:402-8.

7. Pfaffl, M.W. A new mathematical model for relative quantification in real-time rt-pcr. Nucleic. Acids. Res. 2001;29:e45.

8. Hellemans, J., Mortier, G., De Paepe, A., Speleman, F., & Vandesompele, J. Qbase relative quantification framework and software for management and automated analysis of real-time quantitative pcr data. Genome. Biol. 2007;8:R19.
